# (2-Chloro-4-nitro­benzoato)(methanol)triphenyl­tin(IV)

**DOI:** 10.1107/S1600536811011962

**Published:** 2011-04-07

**Authors:** Yip-Foo Win, Chen-Shang Choong, Sie-Tiong Ha, Ching Kheng Quah, Hoong-Kun Fun

**Affiliations:** a Department of Chemical Science, Faculty of Science, Universiti Tunku Abdul Rahman, 31900 Kampar, Perak, Malaysia; b X-ray Crystallography Unit, School of Physics, Universiti Sains Malaysia, 11800 USM, Penang, Malaysia

## Abstract

In the title complex, [Sn(C_6_H_5_)_3_(C_7_H_3_ClNO_4_)(CH_4_O)], the five-coordinate Sn^IV^ atom exists in a trigonal–bipyramidal environment, formed by a monodentate carboxyl­ate group, three phenyl rings and a methanol mol­ecule. The axial sites are occupied by the O atoms of the methanol mol­ecule and the carboxyl­ate group, while the equatorial plane is formed by the C atoms of three phenyl rings. The benzene ring of the 2-chloro-4-nitro­benzoate ligand makes dihedral angles of 66.18 (7), 74.71 (7) and 77.39 (7)° with respect to the three phenyl rings. In the crystal, the mol­ecules are linked *via* inter­molecular O—H⋯O and C—H⋯O hydrogen bonds into a column along the *b* axis.

## Related literature

For general background to and the coordination environment of the title complex, see: Yeap & Teoh (2003 ▶); Szorcsik *et al.* (2006 ▶); Álvarez-Boo *et al.* (2006 ▶). For the stability of the temperature controller used in the data collection, see: Cosier & Glazer (1986 ▶). For bond-length data, see: Allen *et al.* (1987 ▶).
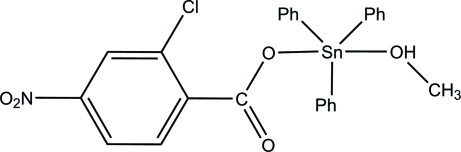


## Experimental

### 

#### Crystal data

[Sn(C_6_H_5_)_3_(C_7_H_3_ClNO_4_)(CH_4_O)]
*M* *_r_* = 582.59
Monoclinic, 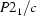

*a* = 9.0287 (3) Å
*b* = 13.5239 (4) Å
*c* = 20.7505 (6) Å
β = 108.894 (1)°
*V* = 2397.19 (13) Å^3^
*Z* = 4
Mo *K*α radiation
μ = 1.22 mm^−1^
*T* = 100 K
0.27 × 0.25 × 0.15 mm

#### Data collection

Bruker SMART APEXII DUO CCD area-detector diffractometer
Absorption correction: multi-scan (*SADABS*; Bruker, 2009 ▶) *T*_min_ = 0.739, *T*_max_ = 0.835
42634 measured reflections
10460 independent reflections
9263 reflections with *I* > 2σ(*I*)
*R*_int_ = 0.023

#### Refinement

*R*[*F* ^2^ > 2σ(*F* ^2^)] = 0.024
*wR*(*F* ^2^) = 0.060
*S* = 1.04
10460 reflections
312 parameters
H atoms treated by a mixture of independent and constrained refinement
Δρ_max_ = 2.82 e Å^−3^
Δρ_min_ = −1.27 e Å^−3^

### 

Data collection: *APEX2* (Bruker, 2009 ▶); cell refinement: *SAINT* (Bruker, 2009 ▶); data reduction: *SAINT*; program(s) used to solve structure: *SHELXTL* (Sheldrick, 2008 ▶); program(s) used to refine structure: *SHELXTL*; molecular graphics: *SHELXTL*; software used to prepare material for publication: *SHELXTL* and *PLATON* (Spek, 2009 ▶).

## Supplementary Material

Crystal structure: contains datablocks global, I. DOI: 10.1107/S1600536811011962/is2695sup1.cif

Structure factors: contains datablocks I. DOI: 10.1107/S1600536811011962/is2695Isup2.hkl

Additional supplementary materials:  crystallographic information; 3D view; checkCIF report

## Figures and Tables

**Table 1:** Hydrogen-bond geometry (Å, °)

*D*—H⋯*A*	*D*—H	H⋯*A*	*D*⋯*A*	*D*—H⋯*A*
O3—H1*O*3⋯O2^i^	0.79 (3)	1.83 (3)	2.6082 (14)	170 (3)
C16—H16*A*⋯O4^ii^	0.93	2.58	3.312 (2)	136

Symmetry codes: (i) 
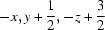
; (ii) 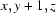.

**Fig. 1.**
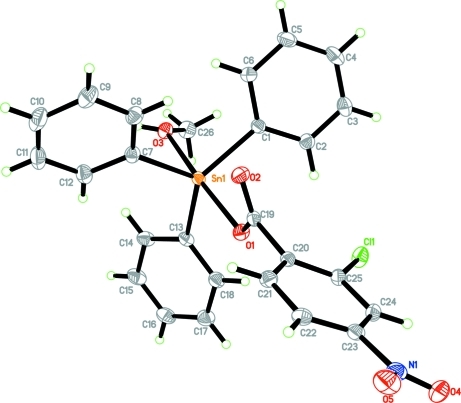
The molecular structure of the title compound, showing 50% probability displacement ellipsoids for non-H atoms.

**Fig. 2.**
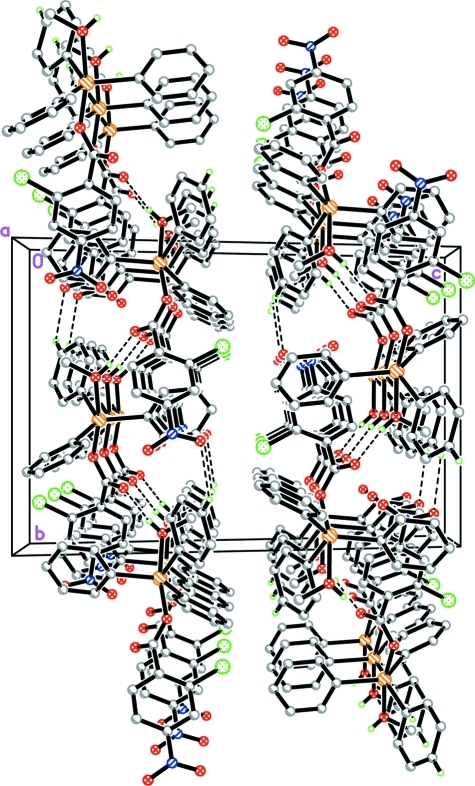
Packing diagram of the title compound, viewed along the a axis. H atoms not involved in hydrogen bonds (dashed lines) have been omitted for clarity.

## supplementary crystallographic information

### Comment

There are a number of well documented literature indicating triphenyltin(IV) carboxylate complexes existing in monomeric five-coordinated structures, e.g. in structures in which the carboxylate anion is bonded to the tin(IV) atom in a monodentate mode and extra coordination being contributed from the coordinating ligands (Yeap & Teoh, 2003; Szorcsik *et al.*, 2006; Álvarez-Boo *et al.*, 2006). The coordinating ligands may be derivatives of coordinating solvents or from pyridine derivatives or amine. Complex (2,6-difluorobenzoato)triphenyltin(IV) showed that the two fluorine atoms at the ortho positions of the benzene fragment while the ethanol molecule was attached to the tin(IV) atom resulting the tin(IV) atom being five-coordinated (Yeap & Teoh, 2003).

The structure of the titled complex (Fig. 1) is similar to (2,6-difluorobenzoato)triphenyltin(IV). The only difference is that the methanol molecule participated in the crystal structure. In the title complex, the five-coordinate tin(IV) (Sn1) atom exists in a trigonal-bipyramidal environment, formed by a monodentate carboxylate group, three phenyl rings and a coordinated methanol molecule. The axial sites are occupied by the O atoms of the methanol and carboxylate [O1—Sn1—O3 = 169.05 (3)°], with the three phenyl rings occupying the equatorial plane. Bond lengths (Allen *et al.*, 1987) and angles are within normal ranges. The benzene ring (C20–C25) of 2-chloro-4-nitrobenzoate ligand makes dihedral angles of 66.18 (7), 74.71 (7) and 77.39 (7)° with respect to the three phenyl rings (C1–C6, C7–C12 and C13–C18). In the crystal structure, the molecules are linked *via* intermolecular O3–H1O3···O2 and C16–H16A···O4 hydrogen bonds (Table 1) into a column along the *b* axis (Fig. 2).

### Experimental

The title complex was obtained by heating under reflux a 1:1 molar mixture of triphenyltin(IV) hydroxide (1.10 g, 3 mmol) and 2-chloro-4-nitrobenzoic acid (0.60 g, 3 mmol) in methanol (50 mL) for 2 h. A clear transparent solution was isolated by filtration and kept in a bottle. After a few days, crystals (1.36 g, 78.2 % yield) were collected (*m.p.*: 119.0-120.0 °C). Analysis for C_26_H_22_NO_5_ClSn: C 53.45, H 3.74, N 2.34%. Calculated for C_26_H_22_NO_5_ClSn: C 53.60, H 3.81, N 2.40%.

### Refinement

H1O3 was located in a difference Fourier map and allowed to refined freely. The remaining H atoms were positioned geometrically and refined using a riding model with C–H = 0.93 or 0.96 Å and *U*_iso_(H) = 1.2 or 1.5 *U*_eq_(C). A rotating-group model was applied for the methyl group. The highest residual electron density peak and the deepest hole are located at 0.57 and 0.53 Å, respectively, from atom Sn1.

### Crystal data

**Table:**

[Sn(C_6_H_5_)_3_(C_7_H_3_ClNO_4_)(CH_4_O)]	*F*(000) = 1168
*M**_r_* = 582.59	*D*_x_ = 1.614 Mg m^−^^3^
Monoclinic, *P*2_1_/*c*	Mo *K*α radiation, λ = 0.71073 Å
Hall symbol: -P 2ybc	Cell parameters from 9909 reflections
*a* = 9.0287 (3) Å	θ = 3.7–37.5°
*b* = 13.5239 (4) Å	µ = 1.22 mm^−^^1^
*c* = 20.7505 (6) Å	*T* = 100 K
β = 108.894 (1)°	Block, yellow
*V* = 2397.19 (13) Å^3^	0.27 × 0.25 × 0.15 mm
*Z* = 4	

### Data collection

**Table:**

Bruker SMART APEXII DUO CCD area-detector diffractometer	10460 independent reflections
Radiation source: fine-focus sealed tube	9263 reflections with *I* > 2σ(*I*)
graphite	*R*_int_ = 0.023
φ and ω scans	θ_max_ = 35.0°, θ_min_ = 1.8°
Absorption correction: multi-scan (*SADABS*; Bruker, 2009)	*h* = −14→13
*T*_min_ = 0.739, *T*_max_ = 0.835	*k* = −21→21
42634 measured reflections	*l* = −33→33

### Refinement

**Table:**

Refinement on *F*^2^	Primary atom site location: structure-invariant direct methods
Least-squares matrix: full	Secondary atom site location: difference Fourier map
*R*[*F*^2^ > 2σ(*F*^2^)] = 0.024	Hydrogen site location: inferred from neighbouring sites
*wR*(*F*^2^) = 0.060	H atoms treated by a mixture of independent and constrained refinement
*S* = 1.04	*w* = 1/[σ^2^(*F*_o_^2^) + (0.023*P*)^2^ + 1.8694*P*] where *P* = (*F*_o_^2^ + 2*F*_c_^2^)/3
10460 reflections	(Δ/σ)_max_ = 0.002
312 parameters	Δρ_max_ = 2.82 e Å^−^^3^
0 restraints	Δρ_min_ = −1.27 e Å^−^^3^

### Special details

**Table:**

Experimental. The crystal was placed in the cold stream of an Oxford Cyrosystems Cobra open-flow nitrogen cryostat (Cosier & Glazer, 1986) operating at 100.0 (1) K.
Geometry. All esds (except the esd in the dihedral angle between two l.s. planes) are estimated using the full covariance matrix. The cell esds are taken into account individually in the estimation of esds in distances, angles and torsion angles; correlations between esds in cell parameters are only used when they are defined by crystal symmetry. An approximate (isotropic) treatment of cell esds is used for estimating esds involving l.s. planes.
Refinement. Refinement of F^2^ against ALL reflections. The weighted R-factor wR and goodness of fit S are based on F^2^, conventional R-factors R are based on F, with F set to zero for negative F^2^. The threshold expression of F^2^ > 2sigma(F^2^) is used only for calculating R-factors(gt) etc. and is not relevant to the choice of reflections for refinement. R-factors based on F^2^ are statistically about twice as large as those based on F, and R- factors based on ALL data will be even larger.

### Fractional atomic coordinates and isotropic or equivalent isotropic displacement parameters (Å^2^)

**Table:**

	*x*	*y*	*z*	*U*_iso_*/*U*_eq_	
Sn1	0.122653 (9)	0.424304 (6)	0.818254 (4)	0.01437 (2)	
Cl1	0.40606 (4)	0.15616 (2)	0.956246 (16)	0.02241 (6)	
O1	0.29055 (11)	0.30392 (7)	0.83558 (5)	0.01747 (16)	
O2	0.11079 (11)	0.20667 (7)	0.76526 (5)	0.01919 (17)	
O3	−0.03497 (11)	0.56098 (7)	0.82164 (5)	0.01848 (16)	
O4	0.73247 (14)	−0.13409 (9)	0.91070 (7)	0.0300 (2)	
O5	0.65369 (15)	−0.16195 (9)	0.80196 (7)	0.0315 (2)	
N1	0.65602 (14)	−0.11287 (9)	0.85191 (7)	0.0221 (2)	
C1	−0.01601 (14)	0.35355 (9)	0.86993 (6)	0.01578 (19)	
C2	0.05009 (15)	0.30813 (10)	0.93321 (6)	0.0186 (2)	
H2A	0.1583	0.3075	0.9535	0.022*	
C3	−0.04454 (18)	0.26366 (11)	0.96633 (7)	0.0228 (2)	
H3A	0.0006	0.2345	1.0088	0.027*	
C4	−0.20606 (18)	0.26288 (11)	0.93602 (8)	0.0256 (3)	
H4A	−0.2691	0.2331	0.9581	0.031*	
C5	−0.27347 (17)	0.30654 (12)	0.87268 (8)	0.0262 (3)	
H5A	−0.3815	0.3053	0.8521	0.031*	
C6	−0.17904 (15)	0.35222 (11)	0.84008 (7)	0.0220 (2)	
H6A	−0.2248	0.3822	0.7980	0.026*	
C7	0.02070 (15)	0.43698 (9)	0.71033 (6)	0.0180 (2)	
C8	−0.09430 (16)	0.37151 (10)	0.67264 (7)	0.0212 (2)	
H8A	−0.1250	0.3196	0.6949	0.025*	
C9	−0.16407 (19)	0.38189 (13)	0.60273 (7)	0.0279 (3)	
H9A	−0.2413	0.3379	0.5786	0.033*	
C10	−0.1169 (2)	0.45913 (14)	0.56922 (8)	0.0311 (3)	
H10A	−0.1608	0.4660	0.5223	0.037*	
C11	−0.0041 (2)	0.52566 (12)	0.60623 (8)	0.0294 (3)	
H11A	0.0261	0.5777	0.5840	0.035*	
C12	0.06386 (17)	0.51509 (10)	0.67604 (7)	0.0229 (2)	
H12A	0.1388	0.5603	0.7003	0.027*	
C13	0.31789 (14)	0.51920 (9)	0.86282 (7)	0.0173 (2)	
C14	0.30934 (16)	0.61928 (10)	0.84459 (8)	0.0217 (2)	
H14A	0.2159	0.6449	0.8156	0.026*	
C15	0.43893 (17)	0.68101 (11)	0.86929 (8)	0.0252 (3)	
H15A	0.4316	0.7472	0.8566	0.030*	
C16	0.57927 (17)	0.64392 (11)	0.91288 (8)	0.0250 (3)	
H16A	0.6659	0.6850	0.9293	0.030*	
C17	0.58899 (17)	0.54502 (12)	0.93165 (8)	0.0249 (3)	
H17A	0.6824	0.5199	0.9610	0.030*	
C18	0.45934 (15)	0.48302 (10)	0.90679 (7)	0.0206 (2)	
H18A	0.4673	0.4169	0.9197	0.025*	
C19	0.24398 (14)	0.22178 (9)	0.80545 (6)	0.01492 (18)	
C20	0.36213 (13)	0.13888 (9)	0.82013 (6)	0.01443 (18)	
C21	0.39401 (15)	0.09388 (10)	0.76536 (6)	0.0179 (2)	
H21A	0.3483	0.1191	0.7217	0.021*	
C22	0.49241 (15)	0.01247 (10)	0.77476 (7)	0.0191 (2)	
H22A	0.5136	−0.0170	0.7382	0.023*	
C23	0.55811 (14)	−0.02348 (9)	0.84060 (7)	0.0172 (2)	
C24	0.53294 (15)	0.02005 (9)	0.89647 (7)	0.0176 (2)	
H24A	0.5799	−0.0050	0.9401	0.021*	
C25	0.43558 (14)	0.10218 (9)	0.88556 (6)	0.01563 (19)	
C26	−0.02762 (18)	0.60801 (11)	0.88443 (7)	0.0240 (2)	
H26A	−0.0895	0.6672	0.8750	0.036*	
H26B	0.0791	0.6244	0.9092	0.036*	
H26D	−0.0675	0.5639	0.9111	0.036*	
H1O3	−0.061 (3)	0.600 (2)	0.7921 (13)	0.042 (7)*	

### Atomic displacement parameters (Å^2^)

**Table:**

	*U*^11^	*U*^22^	*U*^33^	*U*^12^	*U*^13^	*U*^23^
Sn1	0.01494 (4)	0.01250 (4)	0.01648 (4)	−0.00049 (2)	0.00621 (3)	0.00090 (3)
Cl1	0.03009 (15)	0.02303 (13)	0.01545 (12)	0.00816 (11)	0.00921 (10)	0.00142 (10)
O1	0.0181 (4)	0.0137 (4)	0.0201 (4)	0.0010 (3)	0.0055 (3)	−0.0007 (3)
O2	0.0163 (4)	0.0168 (4)	0.0221 (4)	0.0006 (3)	0.0028 (3)	0.0003 (3)
O3	0.0205 (4)	0.0156 (4)	0.0193 (4)	0.0028 (3)	0.0063 (3)	−0.0005 (3)
O4	0.0276 (5)	0.0227 (5)	0.0402 (6)	0.0081 (4)	0.0116 (5)	0.0084 (4)
O5	0.0357 (6)	0.0223 (5)	0.0446 (7)	0.0030 (4)	0.0243 (5)	−0.0055 (5)
N1	0.0202 (5)	0.0152 (4)	0.0354 (6)	0.0010 (4)	0.0152 (4)	0.0017 (4)
C1	0.0150 (4)	0.0148 (5)	0.0181 (5)	−0.0006 (4)	0.0063 (4)	0.0003 (4)
C2	0.0192 (5)	0.0196 (5)	0.0173 (5)	0.0004 (4)	0.0062 (4)	0.0006 (4)
C3	0.0295 (6)	0.0213 (6)	0.0208 (5)	0.0000 (5)	0.0124 (5)	0.0021 (4)
C4	0.0281 (6)	0.0227 (6)	0.0327 (7)	−0.0043 (5)	0.0191 (6)	0.0002 (5)
C5	0.0179 (5)	0.0258 (6)	0.0369 (7)	−0.0027 (5)	0.0117 (5)	0.0017 (6)
C6	0.0164 (5)	0.0232 (6)	0.0254 (6)	−0.0007 (4)	0.0051 (4)	0.0045 (5)
C7	0.0198 (5)	0.0161 (5)	0.0184 (5)	0.0027 (4)	0.0068 (4)	0.0011 (4)
C8	0.0223 (5)	0.0190 (5)	0.0198 (5)	−0.0003 (4)	0.0030 (4)	0.0000 (4)
C9	0.0295 (7)	0.0317 (7)	0.0188 (6)	0.0060 (6)	0.0028 (5)	−0.0015 (5)
C10	0.0347 (8)	0.0397 (8)	0.0186 (6)	0.0145 (7)	0.0080 (5)	0.0054 (6)
C11	0.0379 (8)	0.0268 (7)	0.0282 (7)	0.0095 (6)	0.0173 (6)	0.0111 (5)
C12	0.0274 (6)	0.0193 (5)	0.0246 (6)	0.0022 (5)	0.0121 (5)	0.0034 (5)
C13	0.0170 (5)	0.0158 (5)	0.0211 (5)	−0.0021 (4)	0.0088 (4)	−0.0014 (4)
C14	0.0191 (5)	0.0151 (5)	0.0325 (7)	−0.0016 (4)	0.0104 (5)	−0.0015 (5)
C15	0.0243 (6)	0.0172 (5)	0.0371 (7)	−0.0055 (5)	0.0140 (5)	−0.0038 (5)
C16	0.0229 (6)	0.0256 (6)	0.0282 (6)	−0.0084 (5)	0.0108 (5)	−0.0061 (5)
C17	0.0201 (6)	0.0289 (7)	0.0240 (6)	−0.0043 (5)	0.0049 (5)	−0.0013 (5)
C18	0.0199 (5)	0.0206 (5)	0.0215 (5)	−0.0023 (4)	0.0070 (4)	0.0002 (4)
C19	0.0160 (4)	0.0140 (4)	0.0156 (4)	0.0007 (4)	0.0062 (4)	0.0025 (4)
C20	0.0142 (4)	0.0138 (4)	0.0159 (4)	−0.0004 (4)	0.0057 (4)	0.0007 (4)
C21	0.0168 (5)	0.0217 (5)	0.0156 (5)	0.0012 (4)	0.0058 (4)	−0.0006 (4)
C22	0.0178 (5)	0.0212 (5)	0.0205 (5)	0.0002 (4)	0.0094 (4)	−0.0033 (4)
C23	0.0162 (5)	0.0133 (4)	0.0244 (5)	0.0009 (4)	0.0096 (4)	0.0006 (4)
C24	0.0189 (5)	0.0156 (5)	0.0193 (5)	0.0026 (4)	0.0077 (4)	0.0033 (4)
C25	0.0176 (5)	0.0147 (4)	0.0155 (5)	0.0012 (4)	0.0067 (4)	0.0006 (4)
C26	0.0268 (6)	0.0243 (6)	0.0232 (6)	0.0026 (5)	0.0112 (5)	−0.0036 (5)

### Geometric parameters (Å, °)

**Table:**

Sn1—C1	2.1218 (12)	C10—C11	1.389 (3)
Sn1—C13	2.1339 (12)	C10—H10A	0.9300
Sn1—C7	2.1346 (13)	C11—C12	1.386 (2)
Sn1—O1	2.1737 (9)	C11—H11A	0.9300
Sn1—O3	2.3477 (9)	C12—H12A	0.9300
Cl1—C25	1.7344 (12)	C13—C18	1.3958 (18)
O1—C19	1.2772 (15)	C13—C14	1.4008 (18)
O2—C19	1.2385 (15)	C14—C15	1.3929 (19)
O3—C26	1.4320 (17)	C14—H14A	0.9300
O3—H1O3	0.78 (3)	C15—C16	1.390 (2)
O4—N1	1.2256 (18)	C15—H15A	0.9300
O5—N1	1.2252 (17)	C16—C17	1.388 (2)
N1—C23	1.4709 (17)	C16—H16A	0.9300
C1—C2	1.3965 (17)	C17—C18	1.3959 (19)
C1—C6	1.4001 (17)	C17—H17A	0.9300
C2—C3	1.3944 (19)	C18—H18A	0.9300
C2—H2A	0.9300	C19—C20	1.5091 (16)
C3—C4	1.389 (2)	C20—C25	1.3951 (16)
C3—H3A	0.9300	C20—C21	1.3989 (17)
C4—C5	1.389 (2)	C21—C22	1.3883 (18)
C4—H4A	0.9300	C21—H21A	0.9300
C5—C6	1.393 (2)	C22—C23	1.3892 (19)
C5—H5A	0.9300	C22—H22A	0.9300
C6—H6A	0.9300	C23—C24	1.3831 (18)
C7—C8	1.3957 (18)	C24—C25	1.3888 (17)
C7—C12	1.3978 (19)	C24—H24A	0.9300
C8—C9	1.3889 (19)	C26—H26A	0.9600
C8—H8A	0.9300	C26—H26B	0.9600
C9—C10	1.396 (3)	C26—H26D	0.9600
C9—H9A	0.9300		
			
C1—Sn1—C13	126.30 (5)	C10—C11—H11A	119.7
C1—Sn1—C7	116.22 (5)	C11—C12—C7	120.54 (14)
C13—Sn1—C7	114.92 (5)	C11—C12—H12A	119.7
C1—Sn1—O1	94.17 (4)	C7—C12—H12A	119.7
C13—Sn1—O1	86.84 (4)	C18—C13—C14	118.29 (12)
C7—Sn1—O1	105.92 (4)	C18—C13—Sn1	121.67 (9)
C1—Sn1—O3	82.95 (4)	C14—C13—Sn1	119.91 (9)
C13—Sn1—O3	86.40 (4)	C15—C14—C13	120.90 (13)
C7—Sn1—O3	84.75 (4)	C15—C14—H14A	119.6
O1—Sn1—O3	169.05 (3)	C13—C14—H14A	119.6
C19—O1—Sn1	117.97 (8)	C16—C15—C14	120.21 (14)
C26—O3—Sn1	121.80 (8)	C16—C15—H15A	119.9
C26—O3—H1O3	109.1 (19)	C14—C15—H15A	119.9
Sn1—O3—H1O3	122.4 (19)	C17—C16—C15	119.46 (13)
O5—N1—O4	124.42 (13)	C17—C16—H16A	120.3
O5—N1—C23	117.74 (12)	C15—C16—H16A	120.3
O4—N1—C23	117.83 (12)	C16—C17—C18	120.39 (14)
C2—C1—C6	118.57 (12)	C16—C17—H17A	119.8
C2—C1—Sn1	122.08 (9)	C18—C17—H17A	119.8
C6—C1—Sn1	119.35 (9)	C13—C18—C17	120.76 (13)
C3—C2—C1	120.64 (12)	C13—C18—H18A	119.6
C3—C2—H2A	119.7	C17—C18—H18A	119.6
C1—C2—H2A	119.7	O2—C19—O1	124.55 (11)
C4—C3—C2	120.09 (13)	O2—C19—C20	118.82 (11)
C4—C3—H3A	120.0	O1—C19—C20	116.63 (10)
C2—C3—H3A	120.0	C25—C20—C21	118.55 (11)
C3—C4—C5	119.97 (13)	C25—C20—C19	122.89 (10)
C3—C4—H4A	120.0	C21—C20—C19	118.50 (10)
C5—C4—H4A	120.0	C22—C21—C20	121.44 (11)
C4—C5—C6	119.91 (13)	C22—C21—H21A	119.3
C4—C5—H5A	120.0	C20—C21—H21A	119.3
C6—C5—H5A	120.0	C21—C22—C23	117.76 (11)
C5—C6—C1	120.81 (13)	C21—C22—H22A	121.1
C5—C6—H6A	119.6	C23—C22—H22A	121.1
C1—C6—H6A	119.6	C24—C23—C22	122.79 (11)
C8—C7—C12	118.27 (12)	C24—C23—N1	118.00 (12)
C8—C7—Sn1	121.79 (10)	C22—C23—N1	119.19 (12)
C12—C7—Sn1	119.88 (10)	C23—C24—C25	118.10 (11)
C9—C8—C7	121.64 (14)	C23—C24—H24A	120.9
C9—C8—H8A	119.2	C25—C24—H24A	120.9
C7—C8—H8A	119.2	C24—C25—C20	121.29 (11)
C8—C9—C10	119.19 (15)	C24—C25—Cl1	117.47 (9)
C8—C9—H9A	120.4	C20—C25—Cl1	121.23 (9)
C10—C9—H9A	120.4	O3—C26—H26A	109.5
C11—C10—C9	119.78 (14)	O3—C26—H26B	109.5
C11—C10—H10A	120.1	H26A—C26—H26B	109.5
C9—C10—H10A	120.1	O3—C26—H26D	109.5
C12—C11—C10	120.55 (14)	H26A—C26—H26D	109.5
C12—C11—H11A	119.7	H26B—C26—H26D	109.5
			
C1—Sn1—O1—C19	67.75 (9)	C1—Sn1—C13—C18	70.01 (12)
C13—Sn1—O1—C19	−166.06 (9)	C7—Sn1—C13—C18	−128.94 (11)
C7—Sn1—O1—C19	−51.03 (10)	O1—Sn1—C13—C18	−22.84 (11)
O3—Sn1—O1—C19	142.03 (17)	O3—Sn1—C13—C18	148.55 (11)
C1—Sn1—O3—C26	66.69 (10)	C1—Sn1—C13—C14	−114.27 (11)
C13—Sn1—O3—C26	−60.57 (10)	C7—Sn1—C13—C14	46.78 (12)
C7—Sn1—O3—C26	−176.03 (10)	O1—Sn1—C13—C14	152.88 (11)
O1—Sn1—O3—C26	−8.6 (2)	O3—Sn1—C13—C14	−35.73 (10)
C13—Sn1—C1—C2	−43.07 (12)	C18—C13—C14—C15	0.4 (2)
C7—Sn1—C1—C2	156.10 (10)	Sn1—C13—C14—C15	−175.42 (11)
O1—Sn1—C1—C2	46.07 (11)	C13—C14—C15—C16	−0.2 (2)
O3—Sn1—C1—C2	−123.32 (11)	C14—C15—C16—C17	−0.1 (2)
C13—Sn1—C1—C6	136.78 (10)	C15—C16—C17—C18	0.3 (2)
C7—Sn1—C1—C6	−24.06 (12)	C14—C13—C18—C17	−0.3 (2)
O1—Sn1—C1—C6	−134.09 (10)	Sn1—C13—C18—C17	175.52 (11)
O3—Sn1—C1—C6	56.53 (10)	C16—C17—C18—C13	−0.1 (2)
C6—C1—C2—C3	−0.89 (19)	Sn1—O1—C19—O2	2.35 (16)
Sn1—C1—C2—C3	178.95 (10)	Sn1—O1—C19—C20	−178.31 (7)
C1—C2—C3—C4	1.0 (2)	O2—C19—C20—C25	−121.72 (13)
C2—C3—C4—C5	−0.1 (2)	O1—C19—C20—C25	58.90 (16)
C3—C4—C5—C6	−0.9 (2)	O2—C19—C20—C21	55.28 (16)
C4—C5—C6—C1	0.9 (2)	O1—C19—C20—C21	−124.10 (12)
C2—C1—C6—C5	−0.1 (2)	C25—C20—C21—C22	2.03 (19)
Sn1—C1—C6—C5	−179.91 (11)	C19—C20—C21—C22	−175.10 (11)
C1—Sn1—C7—C8	−25.33 (12)	C20—C21—C22—C23	0.30 (19)
C13—Sn1—C7—C8	171.64 (10)	C21—C22—C23—C24	−1.91 (19)
O1—Sn1—C7—C8	77.66 (11)	C21—C22—C23—N1	176.70 (11)
O3—Sn1—C7—C8	−104.81 (11)	O5—N1—C23—C24	166.13 (12)
C1—Sn1—C7—C12	151.78 (10)	O4—N1—C23—C24	−12.73 (18)
C13—Sn1—C7—C12	−11.26 (12)	O5—N1—C23—C22	−12.54 (18)
O1—Sn1—C7—C12	−105.24 (10)	O4—N1—C23—C22	168.60 (12)
O3—Sn1—C7—C12	72.30 (11)	C22—C23—C24—C25	1.09 (19)
C12—C7—C8—C9	0.7 (2)	N1—C23—C24—C25	−177.53 (11)
Sn1—C7—C8—C9	177.85 (11)	C23—C24—C25—C20	1.37 (19)
C7—C8—C9—C10	0.7 (2)	C23—C24—C25—Cl1	−179.71 (10)
C8—C9—C10—C11	−1.5 (2)	C21—C20—C25—C24	−2.89 (18)
C9—C10—C11—C12	1.0 (2)	C19—C20—C25—C24	174.11 (11)
C10—C11—C12—C7	0.4 (2)	C21—C20—C25—Cl1	178.24 (9)
C8—C7—C12—C11	−1.2 (2)	C19—C20—C25—Cl1	−4.76 (17)
Sn1—C7—C12—C11	−178.43 (11)		

### Hydrogen-bond geometry (Å, °)

**Table:**

*D*—H···*A*	*D*—H	H···*A*	*D*···*A*	*D*—H···*A*
O3—H1O3···O2^i^	0.79 (3)	1.83 (3)	2.6082 (14)	170 (3)
C16—H16A···O4^ii^	0.93	2.58	3.312 (2)	136

Symmetry codes: (i) −*x*, *y*+1/2, −*z*+3/2; (ii) *x*, *y*+1, *z*.
